# Preparation of Alumina Nanorods from Chromium-Containing Alumina Sludge

**DOI:** 10.1186/s11671-017-2160-3

**Published:** 2017-06-08

**Authors:** Xuan Zhang, Bin Deng, Tong Sun, Wei Li, Chang-ping Duan

**Affiliations:** 1 College of Chemistry, Chemical Engineering and Food Safety, Bohai University, Jinzhou, 121013 People’s Republic of China; 2 Jinzhou Petrochemical Engineering Company, Jinzhou, 121001 People’s Republic of China

**Keywords:** Alumina, Nanorods, Doping, Chromium-containing alumina sludge

## Abstract

Alumina nanorods were prepared from chromium-containing alumina sludge, and the effects of doping elements, such as Cr, Fe, and Mg, were researched. The results show that the crystal transformation of alumina is restricted by the doped Cr and facilitated by the doped Fe and Mg, which is transformed from *θ*-Al_2_O_3_ to *α*-Al_2_O_3_ in the calcination process. Meanwhile, the crystal transformation of alumina is strongly restrained by co-doped elements from the chromium-containing alumina sludge. The doped elements change the course of phase structure transformation and slightly transform the chemical bond of the alumina nanorods. The impure elements are doped in the alumina crystal and restrain the crystalline growth of alumina nanorods according to the rules. In the sample prepared from chromium-containing alumina sludge, more Cr and Mg but fewer Fe are doped, and most Cr are existed as Cr(III). It is possible that the Fe-doping is confined by the competition of Cr and Mg. Moreover, the lattice imperfection of alumina is caused by doped ions, such as Cr, Fe, and Mg, and the chemical state of O and Al are affected. The findings by these experiments provide essential information for eliminating pollution and promoting comprehensive utilization of the chromium-containing alumina sludge.

## Background

Low-dimensional nano alumina, such as alumina nanofibers [1–3] and alumina nanorods [4], has superior properties of high strength, high elastic modulus, chemical stability, good thermal insulation performance, and low thermal conductivity [5–9], so it was widely applied in various fields, such as reinforcement for ceramic matrix composites and metal matrix composites and catalyst, catalyst carrier, adsorbents, membrane reactor, coatings, and anode materials [4, 10–15]. However, high cost of production has limited its application. Some authors have reported synthesis methods of low-dimensional nano alumina successfully, mainly including solid-phase method, vapor-phase method [16], and liquid-phase method [17, 18]. Among them, the liquid-phase method is applied widely for its mild reaction condition, homogeneous products, and low cost of production. There were lots of reports about preparation of nano alumina by sol–gel method [5, 19–21], microemulsion method [22], hydrothermal method [23], precipitation method [23], chemical vapor deposition [16], and electrospinning [1, 3, 24, 25]. However, precipitation method is fit for the laboratories and industries because of its low energy consumption, homogeneity of product, and controllable size and shape.

The chromium-containing alumina sludge is a kind of dangerous solid waste, which is produced in the chromium products producing process by non-calcium roasting method. Seven thousand kilograms of the chromium-containing alumina sludge is generated from every ton of chrome product. It is composed of 55 ~ 65% of Al_2_O_3_, 7 ~ 13% of chrome, and few compound of silicon, iron, magnesium, and sodium. The components of the chromium-containing alumina sludge are shown in Table 1, which are provided by manufacturer (CITIC Jinzhou Metal Co., Ltd., China).

**Table 1:** The components of the chromium-containing alumina sludge

Components	Al_2_O_3_	Cr_2_O_3_	SiO_2_	Cr^6+^	Fe_2_O_3_	MgO	Na_2_O	SO_4_^2−^
wt/%	55 ~ 65	1 ~ 5	<4	6 ~ 10	<0.5	<0.036	<0.5	<0.8

As the main hazardous substance, chrome exists in the form of Cr(III) or Cr(VI) in the chromium-containing alumina sludge, and Cr(VI) is considered to be the dominant pollutant due to its carcinogenicity [26]. At present, the solution pollution of the chromium-containing alumina sludge is mainly detoxicated and utilized. The former one transforms Cr(VI) to low toxicity Cr(III), and stocks it as a waste residue. Zhang Dalei [27] noted a pyrolysis method to transform Cr(VI) to Cr(III) using straw. Duan Suhua [28] pointed out that the chromium-containing slag could be treated with industrial alcohol. However, the methods mentioned above not only take up land, but also cause great resource waste. What is more, the secondary pollution may occur unexpectedly. The latter method is to separate and utilize the useful components of the chromium-containing alumina sludge. Xue Wendong [29] reported that the chromium-containing alumina sludge could be used to prepare refractory. However, the above method may be limited due to its low added value. Consequently, some new methods should be put forward to eliminate pollution and promote comprehensive utilization of the chromium-containing alumina sludge, which can not only solve environmental problems but also bring great economic benefit.

In this paper, alumina nanorods are prepared from the chromium-containing alumina sludge by precipitation-calcination method. Meanwhile, in order to research the influence of the single-doping ion on the alumina nanorods, the alumina nanorods with non- or single-doping ion are prepared and characterized. The results will provide technical support for eliminating pollution and promoting comprehensive utilization of the chromium-containing alumina sludge.

## Methods

### Materials

The reagents (e.g., aluminum sulfate octadecahydrate, chromium sulfate, ferric sulfate, magnesium sulfate, sodium hydroxide, sulfuric acid, and sodium dodecyl benzene sulfonate) used in this study were analytically pure chemicals. The chromium-containing alumina sludge was provided by CITIC Jinzhou Metal Co., Ltd. (China). All the solutions were prepared with de-ionized water.

### Treatment of the Chromium-Containing Alumina Sludge

At first, the chromium-containing alumina sludge was washed and filtered by de-ionized water according to the solid–liquid ratio of 1:5 (g/mL). As a result, most of Cr^6+^ compounds were separated from the chromium-containing alumina sludge. Then, the filter cake was dissolved with sulfuric acid according to the solid–liquid ratio of 1:3 (g/mL), and then, the H_2_O_2_ was used to transform the residual Cr^6+^ to Cr^3+^. At last, the chromium-containing alumina sludge acid solution was obtained successfully, and the components were analyzed by chemical titration and visible light spectrophotometer (VIS, 721N, Varian, America) shown in Table 2.

**Table 2:** The components of chromium-containing alumina sludge acid solution

Components	Al^3+^	Cr^3+^	Fe^3+^	Mg^2+^
Concentration (mol/L)	0.50	0.013	0.008	0.005

### Synthesis of Alumina Nanorods

Two moles per liter of NaOH solution and dodecyl benzene sulfonate solution were slowly added into 0.25 mol/L Al_2_(SO_4_)_3_ solution under magnetic stirring at 85 °C, and the pH value of the mixed solution was adjusted to 9.0 with NaOH or H_2_SO_4_ solution. After stirring for 5 h and aging for 20 h, the precipitates were separated and washed several times with de-ionized water and ethyl alcohol. Subsequently, the samples were vacuum-dried at 40 °C for 15 h, and then the precursors were prepared. Finally, the samples were calcined at 250 °C for 1 h, 400 °C for 1 h, 770 °C for 1 h, 900 °C for 1 h, and 1050 °C for 2 h continuously, and then the samples were collected for use. The undoped alumina were prepared from pure Al_2_(SO_4_)_3_ solution, and the ion-doped samples were prepared by the same method as above. Meanwhile, the chlorates of Cr, Fe, and Mg were added in the Al_2_(SO_4_)_3_ solution according to the contents of doping element in the chromium-containing alumina sludge (Table 2), and the Cr-doped, Fe-doped, and Mg-doped alumina were prepared. Using the chromium-containing alumina sludge acid solution as the raw materials, the alumina was named which was prepared from the chromium-containing alumina sludge.

### Characterization of Nano Alumina Rods

The crystalline phases of the samples were characterized by X-ray powder diffraction (XRD) using D/MAX-RB X-ray diffractometer (Rigaku, Japan) with Cu K-radiation in the 2θ range of 10°–70° at a scan rate of 2°/min. Fourier transform infrared spectra (FT-IR) of the samples were characterized using Scimitar 2000 Near FT-IR Spectrometer (Thermo electron, USA), and the spectra were recorded in the range of 4000–400 cm^−1^. The thermal stability of the precursor were examined by thermogravimetric analyzer (TG-DSC, STA449F3, NETZSCH, Germany) with a flow rate of 30 mL/min in air atmosphere and a temperature of 15–1200 °C with a heating rate of 10 °C/min. The morphologies, crystal structure, and element distribution of the samples were examined by field-emission transmission electron microscopy (FETEM, Jem-2100F, JEOL, Japan). The X-ray photoelectron spectroscopy (XPS) spectra of the samples were recorded on XPS (ESCAMABMKLL, VG, UK) equipped with a hemispherical electron analyzer and an Al *Kα* X-ray source.

## Results and Discussion

### XRD Characterization of the Alumina Nanorods

**Fig. 1:**
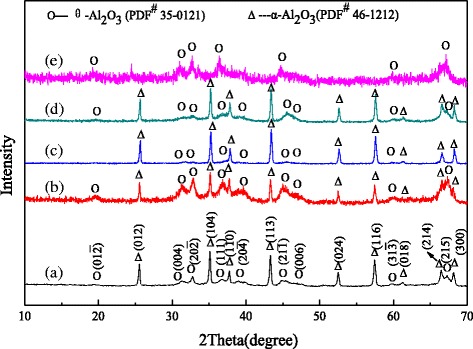
XRD patterns of alumina nanorods doped with different ions: **a** undoped alumina, **b** Cr-doped alumina, **c** Fe-doped alumina, **d** Mg-doped alumina, and **e** the alumina prepared from the chromium-containing alumina sludge

XRD patterns were recorded to confirm the crystal structure of the samples, as shown in Fig. 1. For the undoped alumina nanorods, the XRD results show the existence of different alumina crystalline structures, including corundum (*α*-Al_2_O_3_, syn) (JCPDS No. 46–1212) and aluminum oxide (*θ*-Al_2_O_3_, JCPDS No. 35–0121), and the diffraction peaks of *θ*-Al_2_O_3_ are weaker (Fig. 1 (a)). In general, the alumina are transformed from transition state *θ*-Al_2_O_3_ to steady state *α*-Al_2_O_3_ at 1000 ~ 1200 °C. Compared to the undoped sample, Cr-doped alumina nanorods have relatively stronger peaks of *θ*-Al_2_O_3_ and relatively weaker peaks of *α*-Al_2_O_3_ (Fig. 1 (b)). It means that the crystal transition is restricted by the doped Cr in the calcination process, so less *θ*-Al_2_O_3_ is transformed to *α*-Al_2_O_3_ after calcined at 1050 °C. From Fig. 1 (c), it can be seen that the peaks of *α*-Al_2_O_3_ are stronger and sharper than those in (a) and (b), suggesting the bigger crystal size and better crystallinity. Meanwhile, the peaks of *θ*-Al_2_O_3_ are even weaker, which indicates that the crystal transition is facilitated by the doped Fe. It may be because more *θ*-Al_2_O_3_ is transformed to *α*-Al_2_O_3_ after calcination. Figure 1 (d) shows that the Mg-doped alumina nanorods have relatively stronger and sharper peaks of *α*-Al_2_O_3_ and relatively weaker peaks of *θ*-Al_2_O_3_. It is suggested that the sample contains more *α*-Al_2_O_3_ and less *θ*-Al_2_O_3_, which may be due to that the doped Mg promotes crystal transition of alumina in the calcination process. For the alumina nanorods prepared from chromium-containing alumina sludge, the peaks of *α*-Al_2_O_3_ nearly disappear, while the peaks of *θ*-Al_2_O_3_ become stronger but not sharp enough (Fig. 1 (e)). It is indicated that the *θ*-Al_2_O_3_ has poor crystallinity and smaller crystal size. This could be because more impurity elements of the chromium-containing alumina sludge are doped in the alumina, and the crystal transition of alumina are restricted in the calcination process. So, the *θ*-Al_2_O_3_ is rarely transformed to *α*-Al_2_O_3_.

### FT-IR Spectra of the Alumina Nanorods

**Fig. 2:**
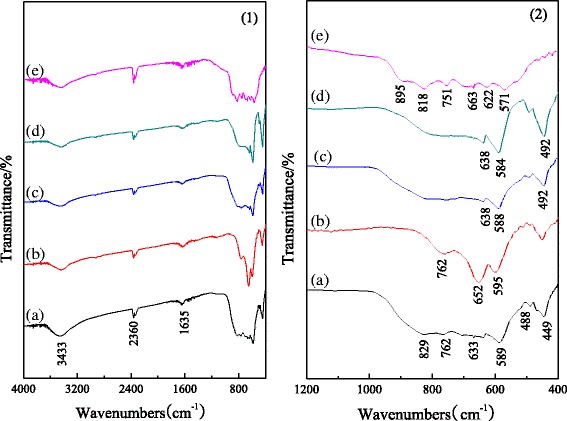
FT-IR spectra of nano alumina rods doped with different ions: **a** undoped alumina, **b** Cr-doped alumina, **c** Fe-doped alumina, **d** Mg-doped alumina, and **e** the alumina prepared from the chromium-containing alumina sludge

FT-IR spectra of alumina nanorods in the range of 4000–400 cm^−1^ are depicted in Fig. 2 [27]. The absorption peaks at 3500–3300 and 1635 cm^−1^ that appear in all the spectra are attributed to the stretching vibration of non-chemical bond association of OH groups and H–O–H bending vibrations, respectively, indicating that the pore water and adsorbed water exist in the samples [30]. The peaks at 2360 cm^−1^ are attributed to the presence of carbon dioxide. Figure 2 (2) shows the fingerprint region of FT-IR spectra of the samples. As shown in Fig. 2 (2a), for the undoped sample, the peaks at 829, 589, and 449 cm^−1^ are attributed to AlO_6_ vibrations, indicating the formation of *α*-Al_2_O_3_ [1]. Meanwhile, the peaks at 762 cm^−1^ are attributed to the bending vibration of Al–O–Al, and the ones at 663 and 488 cm^−1^ are attributed to the stretching vibrations and bending vibration of Al–O, respectively, indicating the formation of *θ*-Al_2_O_3_. Figure 2 (2b) shows that the peaks of *α*-Al_2_O_3_ are weaker than those in Fig. 2(2a), indicating that the Cr-doped prevents the formation of *α*-Al_2_O_3_ in the calcination process. For the Fe- and Mg-doped alumina, the peaks of *θ*-Al_2_O_3_ become weaker, and the peaks of *α*-Al_2_O_3_ have very little change (Fig. 2 (2c,d)). Comparing to Fig. 2(2a), the peaks have redshifted or blueshifted slightly, illustrating that the doped Fe and Mg benefit to the growth of *α*-Al_2_O_3_ and transform the chemical bond of the alumina nanorods slightly. Fig. 2 (2e) is the fingerprint region of FT-IR spectra of the alumina nanorods prepared from the chromium-containing alumina sludge. The peaks below 500 cm^−1^ disappear, indicating that there is no *α*-Al_2_O_3_ in the samples. Moreover, the peaks at 900–500 cm^−1^ are dispersed, which could be result of vibrations of M–O and M–O–M (M is Al or the doped element of alumina from the chromium-containing alumina sludge). The above results are in accordance with the XRD results.

### TG-DSC of Alumina Nanorods

**Fig. 3:**
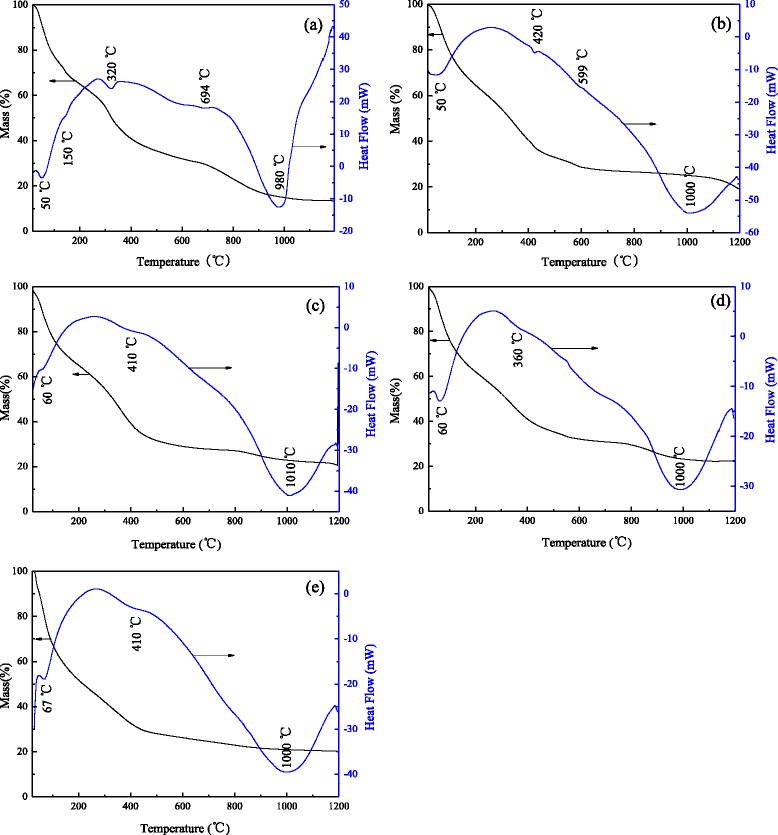
TG and DSC of the nano alumina rod precursors doped with different ions: **a** undoped alumina, **b** Cr-doped alumina, **c** Fe-doped alumina, **d** Mg-doped alumina, and **e** The alumina prepared from the chromium-containing alumina sludge

Thermogravimetric analyzer (TG) and differential scanning calorimetry (DSC) curves of the alumina nanorod precursors are shown in Fig. 3. The XRD results indicate that the alumina nanorod precursor is AlO(OH) (JCPDS No. 49–0133). As shown in Fig. 3a, in the air, only three stages can be seen in the undoped sample. Below 250 °C, about 40% mass loss on the TG curve and a corresponding endothermic peaks at 50 and 150 °C on the DSC curve are associated with moisture evaporation and adsorbed water desorption. The second stage is between 250 and 730 °C, with a total mass loss of about 35% and two endothermic peaks are at 320 and 694 °C. At the temperature of 320 °C, the endothermic peak is due to the transformation of AlO(OH) to amorphous Al_2_O_3_. Meanwhile, the weak endothermic peak at 694 °C is attributed to the transformation of amorphous Al_2_O_3_ to *θ*-Al_2_O_3_. In the third stage above 730 °C, there are a small mass loss and a strong endothermic peak at 980 °C, which is mainly the result of the transformation of *θ*-Al_2_O_3_ to *α*-Al_2_O_3_. Compared to the undoped sample, metal ion doping makes the endothermic peaks shift. Figure 3b–e shows that the endothermic peaks are shifted to higher temperature and became widened. It may be because that the doped ions change the course of phase structure transformation, so the transformation degree of *θ*-Al_2_O_3_ to *α*-Al_2_O_3_ is different for each sample. The results agree with the XRD and FT-IR.

### TEM, SAED, and HRTEM Images of Alumina Nanorods

**Fig. 4:**
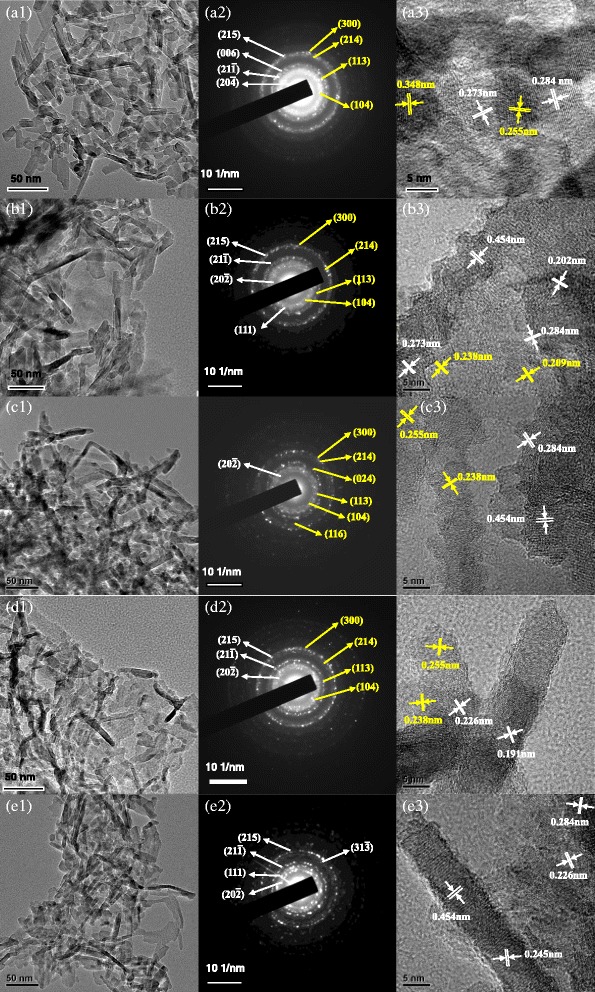
TEM, SAED, and HRTEM of alumina nanorods doped with different ions: **a** undoped alumina, **b** Cr-doped alumina, **c** Fe-doped alumina, **d** Mg-doped alumina, and **e** the alumina prepared from the chromium-containing alumina sludge. *(1)* TEM; *(2)* SAED; *(3)* HRTEM

Figure 4 gives the TEM, selected area electron diffraction (SAED), and high-resolution transmission electron microscopy (HRTEM) results. As shown in Fig. 4 (a1–a3), the undoped alumina are dispersive nanorods with a diameter of 4–6 nm and length of 20–60 nm. Meanwhile, the (215), (006), (21 $\overline{1}$), and (20 $\overline{4}$) planes are in accordance with *θ*-Al_2_O_3_ (JCPDS No. 35–0121), and the (300), (214), (113), and (104) planes are associated with *α*-Al_2_O_3_ (JCPDS No. 46–1212). Furthermore, the observed interplanar distance of 0.273 and 0.284 nm could be assigned to the (20 $\overline{2}$) and (004) planes of *θ*-Al_2_O_3_, and the lattice spacing of 0.255 and 0.348 nm could be corresponded to the (104) and (012) planes of *α*-Al_2_O_3_. Comparing to the undoped sample, the Cr-doped sample is nanorods with a diameter of 4–6 nm and length of 50–120 nm (Fig. 4 (b1)). Figure 4 (b2) shows that the (215), (21 $\overline{1}$), (20 $\overline{2}$), and (111) planes are in accordance with *θ*-Al_2_O_3_, and the (300), (214), (113), and (104) planes are in accordance with *α*-Al_2_O_3_. As shown in Fig. 4 (b3), the interplanar distance of 0.202 nm, 0.273 nm, 0.284 nm, and 0.454 nm are assigned to the (21 $\overline{1}$), (20 $\overline{2}$), (004), and (10 $\overline{2}$) planes of *θ*-Al_2_O_3_, and the interplanar distance of 0.209 and 0.238 nm are assigned to the (113) and (110) planes of *α*-Al_2_O_3_. Figure 4 (c1) shows that the Fe-doped sample is the mixture of nanorods with a diameter of 5–10 nm and length of 30–100 nm and nano particles about 10 nm. Figure 4 (c2) shows that the (20 $\overline{2}$) planes are in accordance with *θ*-Al_2_O_3_, and the (300), (214), (024), (113), (104), and (116) planes are in accordance with *α*-Al_2_O_3_, it is in accordance with the XRD results. Meanwhile, the observed interplanar distance of 0.284 and 0.454 nm are assigned to the (004) and (10 $\overline{2}$) planes of *θ*-Al_2_O_3_, and the interplanar distance of 0.238 and 0.255 nm are assigned to the (110) and (104) planes of *α*-Al_2_O_3_ (Fig. 4 (c3)).

As shown in Fig. 4 (d1–d3), the Mg-doped sample is well-dispersed nanorods with a diameter of 5–10 nm and length of 20–50 nm, and nano particles about 10 nm exist simultaneously. The SAED results show that the (215), (21 $\overline{1}$), and (20 $\overline{2}$) planes are in accordance with *θ*-Al_2_O_3_, and the (300), (214), (113), and (104) planes are in accordance with *α*-Al_2_O_3_. The HRTEM results show that the observed interplanar distance of 0.226 and 0.191 nm are assigned to the (20 $\overline{4}$) and (006) planes of *θ*-Al_2_O_3_, and the interplanar distance of 0.255 and 0.238 nm are assigned to the (104) and (110) planes of *α*-Al_2_O_3_. Figure 4 (e1–e3) shows that the sample prepared from chromium-containing alumina sludge is well-dispersed nanorods with a diameter of 4–6 nm and length of 50–100 nm, and nano particles about 5–10 nm exist simultaneously. The SAED and HRTEM results show that (215), (111), (21 $\overline{1}$), (31 $\overline{3}$), and (20 $\overline{2}$) planes are in accordance with *θ*-Al_2_O_3_, and the observed interplanar distance of 0.226, 0.245, 0.284, and 0.454 nm are assigned to the (20 $\overline{4}$), (111), (004), and (10 $\overline{2}$) planes of it. However, there are no planes in accordance with *α*-Al_2_O_3_. As a result, the undoped alumina nanorods are well-dispersive than the others, and the particles have regular shape. It may be that the impurity elements are doped in the alumina crystal and restrained the crystalline growth of alumina nanorods according to the rules. So, the shapes and dispersibilities of the alumina nanorods are affected by the doped elements.

### EDS Characterization of Alumina Nanorods Precursor Doped with Different Ions

The EDS results reveal that Cr, Fe, and Mg are doped in the alumina nanorods precursor with molar amount of 2.06, 0.99, and 0.58%, respectively (Table 3). This doping amount is close to the addition dosage of impurity element (Table 2), indicating that most of impurity elements are doped in the alumina nanorod precursor. Meanwhile, for the sample prepared from chromium-containing alumina sludge, the doped molar amount of Cr, Fe, and Mg are 2.11, 0.14, and 0.96%, respectively. The results suggest that most of Cr and Mg are doped in the sample, but small amount of Fe are doped in it. It is possible that the doping of Fe is confined by the competition of Cr and Mg.

**Table 3:** The EDS results of aluminum nanorods precursors doped with different ions

The samples	Al (At%)	Cr (At%)	Fe (At%)	Mg (At%)	Total (At%)
Cr-doped alumina nanorods	97.94	2.06	–	–	100.00
Fe-doped alumina nanorods	99.01	–	0.99	–	100.00
Mg-doped alumina nanorods	99.42	–	–	0.58	100.00
The alumina nanorods prepared from chromium-containing alumina sludge	96.79	2.11	0.14	0.96	100.00

### XPS Characterization of Nanometer Alumina Fibers Doped with Different Ions

**Fig. 5:**
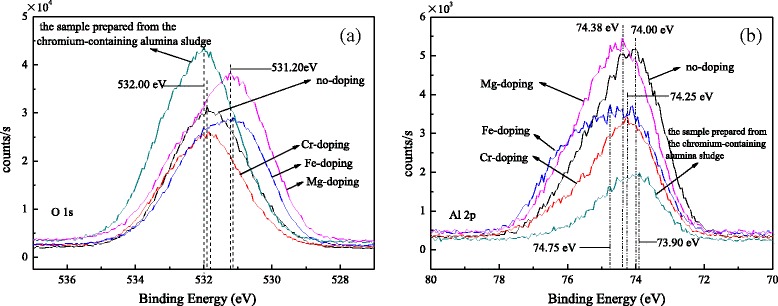
XPS spectra of **a** O1*s* and **b** Al 2*p* for alumina nanorods doped with different ions

Figure 5 shows the XPS spectra of O1*s* and Al 2*p*. As shown in Fig. 5a, the peaks at 531.90, 531.85, 531.15, 531.20, and 532.00 eV are attributed to the undoped, Cr-doped, Fe-doped, and Mg-doped alumina nanorods and the sample prepared from the chromium-containing alumina sludge, respectively. The peaks are assigned to O^2−^ of the Al_2_O_3_ [31]. Figure 5b shows the peaks at 74.00, 74.25, 74.75, 74.38, and 73.90 eV of Al 2*p* are attributed to the above samples, respectively. The peaks are ascribed to Al^3+^ of the Al_2_O_3_. Meanwhile, the good symmetries of curve are proved by Gaussian fitting, indicating that less other oxygen and aluminum are formed in the samples. The O1*s* binding energy (BE) of undoped and Cr-doped alumina nanorods and the sample prepared from the chromium-containing alumina sludge are nearly and are higher than those of Fe-doped and Mg-doped samples. The order of the smallest O1s BE goes as follows: Fe-doped, Mg-doped, Cr-doped, undoped alumina nanorods, and the sample prepared from the chromium-containing alumina sludge. However, the Al *2p* BE is in contrast. XRD results show that more transient state *θ*-Al_2_O_3_ are in the undoped and Cr-doped alumina nanorods and the sample prepared from the chromium-containing alumina sludge, and more *α*-Al_2_O_3_ are in the Fe-doped and Mg-doped alumina nanorods. Due to the coordination form [AlO_4_] for *θ*-Al_2_O_3_ and [AlO_6_] for *α*-Al_2_O_3_, it is possible that the O1*s* BE of [AlO_6_] is bigger and Al 2*p* BE is smaller than that of [AlO_4_]. Moreover, the lattice imperfections of Al_2_O_3_ are caused by Cr, Fe, and Mg ions enter into Al_2_O_3_ lattice. So, the chemical states of O and Al are affected by the lattice defect, and the binding energy is changed.

**Fig. 6:**
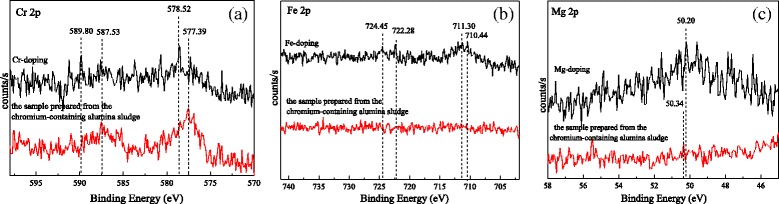
XPS spectra of **a** Cr^3+^ 2*p*, **b** Fe^3+^ 2*p*, and **c** Mg^2+^ 2*p*

Figure 6 presents the XPS spectra of the doping ion. As shown in Fig. 6a, the peaks at 589.80 and 578.52 eV are assigned to Cr *2p*_1/2_ and Cr *2p*_3/2_ of Cr(VI), and the peaks at 587.53 and 577.39 eV are assigned to Cr *2p*_1/2_ and Cr *2p*_3/2_ of Cr(III). It shows that the Cr are existed in the Cr-doped alumina nanorods as Cr(VI) and Cr(III). However, most Cr are existed as Cr(III) in the sample prepared from the chromium-containing alumina sludge. It indicates that the part of Cr(III) are oxidized in calcination process in the Cr-doped sample, but less Cr(III) are oxidized in the samples prepared from the chromium-containing alumina sludge. For the samples prepared from the chromium-containing alumina sludge, because chemical bond combination of Cr–O and the impurity metallic element are formed, the electrode potential of Cr^6+^/Cr^3+^ is increased at high temperature, therefore few Cr(VI) in the sample. As shown in Fig. 6b, the peaks at 724.45 and 711.30 eV are assigned to Fe *2p*_1/2_ and Fe *2p*_3/2_ of Fe_2_O_3_, and 722.38 and 710.44 eV are assigned to Fe *2p*_1/2_ and Fe *2p*_3/2_ of Fe_3_O_4_. The results show that Fe are existed in the Fe-doping sample as Fe(II) and Fe(III). It is suggested that Fe element is entered into the lattice of alumina precursor and take place at the lattice aluminum during the synthesis. At the subsequent calcination process, a little Fe(III) is reduced to Fe(II) by reducing substance in the air. However, there are no peaks of Fe in the sample prepared from the chromium-containing alumina sludge, due to few Fe in the sample (Table 3). As shown in Fig. 6c, the peaks at 50.20 to 50.34 eV are assigned to Mg 2*p* of MgO, suggesting that Mg is existed in the Mg-doped sample as MgO. However, the peak of Mg *2p* is very weak in the sample prepared from the chromium-containing alumina sludge. It is possible that Mg content is seldom. The results agree with the EDS. According to the results of XRD, FT-IR, and XPS, it is illustrated that the lattice imperfection of single-element doping samples is formed due to the impurities of the metallic element entering into the lattice of alumina. However, because of the competition of multiple elements, more Cr is entered into the lattice of alumina prepared from the chromium-containing alumina sludge, and few Fe and Mg elements are entered.

## Conclusions

In summary, the impurity elements were doped in alumina nanorods, such as Cr, Fe, and Mg. The crystal transformation of alumina is restricted by the doped Cr and facilitated by the doped Fe and Mg, which is transformed from *θ*-Al_2_O_3_ to *α*-Al_2_O_3_ in the calcination process. Furthermore, the crystal transformation of alumina is strongly restrained by co-doped elements from the chromium-containing alumina sludge. The course of phase structure transformation, chemical bond, microstructure, and the chemical state of O and Al of the alumina nanorods are affected by the doped elements. In the sample prepared from chromium-containing alumina sludge, the Fe-doping is confined by the competition of Cr and Mg. This study suggests that alumina nanorods may be prepared from chromium-containing alumina sludge to reduce costs and eliminate pollution.

## Abbreviations

BE: Binding energy
DSC: Differential scanning calorimetry analysis
EDS: Energy-dispersive spectrometer
FETEM: Field-emission transmission electron microscopy
FT-IR: Fourier transform infrared spectra
HRTEM: High-resolution transmission electron microscopy
SAED: Selected area electron diffraction
TEM: Transmission electron microscopy
TG: Thermogravimetric analyzer
XPS: X-ray photoelectron spectroscopy
XRD: X-ray powder diffraction
